# Prevalence of dyslipidemia and associated risk factors among adult residents of Shenmu City, China

**DOI:** 10.1371/journal.pone.0250573

**Published:** 2021-05-07

**Authors:** Huiping Gao, Haiying Wang, Guangliang Shan, Rui Liu, Haiyuan Chen, Shengli Sun, Yonglin Liu

**Affiliations:** 1 Shenmu Hospital Affiliated to Northwest University, Shenmu City Hospital, Shenmu, Shaanxi province, China; 2 Institute of Basic Medicine, Chinese Academy of Medical Sciences, Beijing, China; Weill Cornell Medical College in Qatar, QATAR

## Abstract

**Objective:**

Dyslipidemia is a leading risk factor for cardiovascular and cerebrovascular diseases. By collecting the blood lipid profiles among adult residents of Shenmu City in Shaanxi Province, China, we aim to assess and elucidate the prevalence and risk factors of dyslipidemia in this city.

**Method:**

Stratified multistage sampling was used to survey 4,598 permanent adult residents in five areas of Shenmu (2 communities in the county seat, 2 in the southern area and 2 in the northern area) from September 2019 to December 2019. Questionnaire surveys and physical examinations were conducted. Data were analyzed using SPSS software version 26.0.

**Results:**

The average level of total cholesterol (TC) is 4.47mmol/L, that of triglyceride (TG) 1.32mmol/L, high-density lipoprotein cholesterol (HDL-C) 1.27mmol/L, apolipoprotein A1 (ApoA1) 1.44g/L, low-density lipoprotein cholesterol (LDL-C) 2.7mmol/L and apolipoprotein B (ApoB) 0.97g/L. The prevalence of hypercholesterolemia (HTC), hypertriglyceridemia (HTG), low high-density lipoprotein (HDL-C) and high low-density lipoprotein (LDL-C) is 22.4%, 33.3%, 14.5%, and 5.81%, respectively, and the overall prevalence of dyslipidemia is 48.27%. Furthermore, blood lipid levels and prevalence of dyslipidemia vary by region, age, gender, occupation and educational level. Nine risk factors of dyslipidemia were identified, which are living in county seat or northern industrial area, increasing age, male, overweight or obesity, abdominal obesity, smoking, hypertension, abnormal glucose metabolism (pre-diabetes or diabetes) and hyperuricemia.

**Conclusion:**

The blood lipid levels and dyslipidemia prevalence of adults in Shenmu City are higher comparing to national averages of China. Combining risk factors of dyslipidemia, early detection and public health interventions are necessary in high-risk population for associated cardiovascular and cerebrovascular diseases prevention.

## Introduction

Cardiovascular and cerebrovascular diseases are becoming more and more prevalent, and the leading causes of death for Chinese people [1]. Dyslipidemia, either one or a combination of elevated total cholesterol, high LDL-C, low HDL-C, and elevated triglyceride [2], is one of the primary risk factors for atherosclerotic cardiovascular and cerebrovascular diseases [3]. In particular, increased levels of blood lipid profiles exacerbate the incidence of atherosclerosis, which is recognized as the primary risk factor for stroke, peripheral vascular, and heart diseases [4].

LDL-C and HDL-C play important roles in regulating the transportation of cholesterol in our body [5]. Abnormal level of LDL-C is complicated in an increased risk of atherosclerotic cardiovascular disease due to the buildup of plaques within the arteries [2]. However, HDL-C is recognized to protect blood vessel against atherosclerosis through removing cholesterol from the body [6]. Therefore, the imbalance between them can cause the risk of myocardial infarction and stroke.

Over the past decades, the rapid economic development of Shenmu City and the improvement of living conditions mostly contributed to the increase of morbidity and mortality of cardiovascular and cerebrovascular diseases [7]. Several risk factors, including a diet high in saturated fats, sedentary lifestyle, smoking, and obesity remarkably increase the incidence of dyslipidemia that aggravates the development of atherosclerosis [8].

Currently, there is no study showing the prevalence and factors associated with dyslipidemia among the adult population in Shenmu City, China. In this study, we aim to establish a clinical database of blood lipids in Shenmu City, and assess the prevalence of dyslipidemia and its risk factors.

## Materials and methods

### Study design and population

A community-based cross-sectional study was conducted among 4,598 adult residents in Shemu City from September 2019 to December 2019. The method of stratified multistage sampling was performed, the study randomly selected two communities (Linzhou and Yingbin Road community) among the six communities in the county seat, and two towns (Hejiachua and Langanbu towns in the southern area and Daliutang town and Jinjie town in the northern area) among the 14 towns in Shenmu (the city is divided into the northern industrial area and the southern rural area according to GDP, location and landscape, mining area or not, vegetation and desert, and the influence on geological environment caused by mining). Being an adult aged 18 years and above in the city were considered as the inclusion criteria. Five thousand households were selected using a simple random sampling technique from each community and town. Likewise, a single eligible participant was selected using a lottery method from each selected household.

### Data collection and quality control

There are over one hundred research assistants, ten medical laboratory technologist, and one supervisor in the data collection team, which was trained how to conduct interviews, anthropometric measurements, and how to collect and handle the blood sample.

The questionnaire was jointly designed by the Institute of Basic Medicine of the Chinese Academy of Medical Sciences, the Department of Allergy of Beijing Union Hospital and Shenmu City Hospital, with an online version also compiled. The survey was conducted in a face-to-face manner by qualified research assistants. The contents of the questionnaire include general information (demographics, use of the addictive, social environment, past medical history), family information, family history, and drug treatment. The questionnaire was in the local language (Chinese) for participants, and it was also translated to English (Supplement Material). E-questionnaires were stored at cloud services and were reexamined. Before the data collection, all the tools were pre-tested twice to check the completeness, consistency, sensitivity, and applicability and ratified accordingly.

Physical examinations including height, weight, blood pressure, waist circumference, routine blood and urine tests, glycolipid, neck B ultrasound and heart color ultrasound, were conducted at Shenmu City Hospital and Jinjie Town Hospital and Daliuta Town Hospital, while laboratory tests were all carried out at Shenmu City Hospital.

Height was measured by a stadiometer to the nearest 0.1 cm without shoes. Weight was measured using a digital scale to the nearest 0.1 kg with light closes and without shoes. The validation of the weighing scale was checked using a known weight before each measurement. Waist circumference was determined to the nearest 0.1 cm at the midway between the lowest costal margin, midclavicular line, and the anterior superior iliac spine. Triplicate measurement of blood pressure was taken after 5 minutes of rest, and the average systolic blood pressure and diastolic blood pressure were used. All anthropometric measurements were collected in triplicate and the mean values were used for analysis. All measurement data were collected using standardized techniques and calibrated equipment.

### Blood samples collection and laboratory analysis

After the face-to-face interviews and anthropometric measurements, participants were appointed to the next morning (8:30 am–9:30 am) at our assigned hospitals to collect fasting blood. Around 5 ml of blood sample was collected after overnight fasting to determine the contents of total cholesterol (TC), triglyceride (TG), apolipoprotein A1 (Apo A1), apolipoprotein B (Apo B), high-density lipoprotein-cholesterol (HDL-C) and low-density lipoprotein-cholesterol (LDL-C). After clotting for 30 minutes, the blood sample was centrifuged for 10 minutes at 3500 rpm. Around 2.0 ml of pure serum sample was transferred to the barcoded storage tubes and stored at 4 °C. The sample was measured within 2 hours by Cobas c 702 module (Roche Diagnostics) for clinical chemistry analysis.

### Diagnostic criteria

Dyslipidemia: TC≥6.2mmol/L, TG≥2.3mmol/L or HDL-C<1.0 mmol/L, according to the Guidelines for Prevention and Treatment of Dyslipidemia in Chinese Adults (2016 revised version) [3]; the blood lipid level is defined as high if LDL-C≥4.1 mmol/L. Body Mass Index (BMI): <18.5kg/m^2^ is defined as underweight, 18.5≤BMI<24kg/m^2^ is defined as normal, 24≤BMI<28kg/m^2^ overweight, and ≥28 categorized as obese [9]; Abdominal obesity: waist size≥90cm for men, ≥85cm for women [10]; Hypertension: systolic blood pressure≥140 mmHg and or diastolic blood pressure≥90 mmHg or previous diagnosis [11]; Prediabetes: fasting blood glucose 6.1~<7 mmol/L; Diabetes: fasting glucose ≥7.0 mmol/L or previous diagnosis [12].

### Ethical issues

Ethical clearance and approval were obtained from the Ethical Committees of Shenmu Hospital Affiliated to Chinese Northwest University. All participants were informed of what is expected from them and their rights. Written informed consent was obtained from each participant. Illiterate participants put their fingerprint as a signature in the written consent form voluntarily after data collectors read the information. Participants with abnormal lipid profiles were linked to their nearby healthcare facilities for further investigation, counseling, and treatment. Video, sound records and photo were saved. This study was approved by the ethics committee of Shenmu City Hospital (no.: sm004), and all subjects signed informed consent form.

### Statistical analysis

SPSS 26.0 statistical software was used. Continuous variable blood lipid levels were described by mean and standard deviation, and the gender differences of blood lipid level were analyzed by Student’s t-test. The differences by region, age group, occupation and educational level were analyzed by variance analysis and compared in pairs. Counts of dyslipidemia were described by rate, chi-square test was used to compare differences between groups; and multivariate logistic regression was performed to analyze the risk factors of dyslipidemia. Tables were made using excel and R language. Differences in mean values were considered significant at p < 0.05.

## Results

### General situation

**Fig 1:**
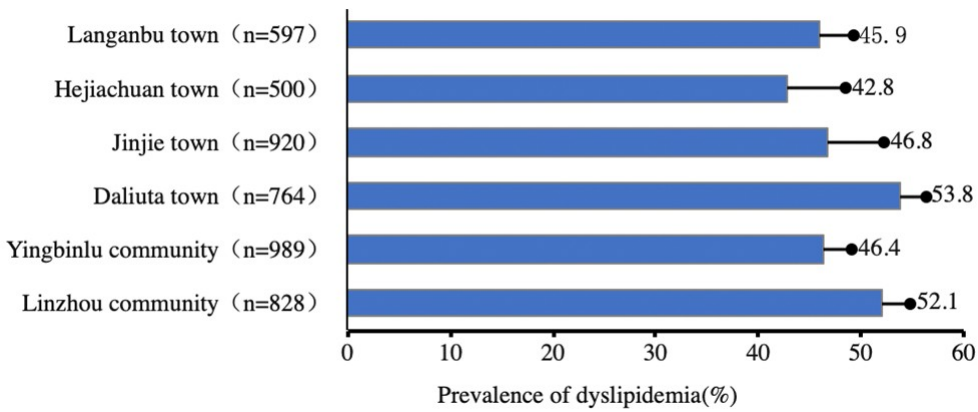
Distribution of sampling points and dyslipidemia.

In this study, 4,706 people were surveyed, and the response rate was 94.1%. After excluding those without a laboratory blood lipid test result (n = 108), 4,598 subjects were included in the analysis. Among the 4,598 respondents, 1,817 live in county, 1,097 in the southern rural area, and 1,684 in northern industrial area. The number of people in each sampling point and the overall prevalence of dyslipidemia were shown in Fig 1. The average age of the respondents is 47.43±11.31 years, of which 1,840 are men, whose average age is 48.59±11.68 years, and 2,758 are women, whose average age is 46.65±10.98 years. Among the respondents, 4,592 are ethnic Han people, accounting for 99.87% of the total. 4,258 respondents, 92.60% of the total, were born in Shenmu City, and 4,567 people now live in Shenmu City, accounting for 99.33% of the total. Education background: primary school or below (2,328 people), secondary school or secondary vocational school (1,695 people), junior college or above (575). Marital status: 4,292 are married, accounting for 93.34%.

### Average value of blood lipids

The average TC level of the respondents is 4.54 mmol/L, the average TG is 1.66 mmol/L. The average HDL-C and Apo A1 are 1.37mmol/L and 1.47g/L, respectively. The average LDL-C and Apo B are 2.76 mmol/L and 0.99g/L, respectively.

Blood lipid levels in different groups of respondents: (1) Except for LDL-C, the differences in the levels of TC, TG, HDL-C, Apo A1 and Apo B are all statistically significant among different regions. The TC and TG levels in the county seat and northern industrial area are significantly higher than those of the southern rural area, and the lowest HDL-C level was observed in the southern rural area. (2) Differences in blood lipid levels between different age groups are statistically significant. The blood lipid levels increased with age, but the levels of TC, LDL-C and Apo B were not enhanced further after 60 years old. Moreover, the lowest HDL-C level was found in people aged 30–49, but not 18–29. (3) There is no significant difference in TG level between people of different occupations, while the differences of the other blood lipid levels are statistically significant. All levels of blood lipids except TG are significantly higher in the group of Agriculture/Forestry/Animal husbandry workers compared with the other two groups. (4) In the term of education background, the differences in blood lipids levels are significant. The lower the educational level, the higher the TC, LDL-C and Apo B levels. TG level is higher in the group with an educational level below secondary vocational school, while HDL-C level is lower in the group with a degree of secondary school or secondary vocational school or above. (5) The differences in blood lipid levels between men and women (p<0.05) have statistical significance, that is for men HDL-C and Apo A1 levels are lower, while levels of TC, TG, LDL-C and Apo B are higher (Table 1).

**Table 1:** Mean value of different blood lipids (Mean±SD).

Item	Blood lipid level (mmol/L or g/L)					
	TC	TG	Apo A1	Apo B	HDL-C	LDL-C
Area						
County seat	4.58±0.94	1.72±1.43	1.45±0.28	1.00±0.27	1.37±0.44	2.76±0.80
Northern area (industrial)	4.60±0.93	1.71±1.31	1.49±0.30	0.96±0.28	1.43±0.62	2.74±0.82
Southern area (rural)	4.39±0.88	1.48±0.97	1.47±0.24	1.02±0.28	1.27±0.31	2.81±0.79
F/Welch F	21.533	20.564	8.578	16.236	48.115	2.625
p	<0.001	<0.001	<0.001	<0.001	<0.001	0.073
Age						
18~29	4.01±0.81	1.28±1.09	1.39±0.28	0.81±0.25	1.41±0.51	2.31±0.71
30~39	4.21±0.81	1.49±1.11	1.41±0.25	0.91±0.26	1.32±0.43	2.53±0.71
40~49	4.52±0.90	1.69±1.29	1.46±0.27	0.99±0.26	1.33±0.45	2.76±0.77
50~59	4.76±0.92	1.81±1.45	1.51±0.28	1.05±0.27	1.39±0.52	2.92±0.81
≥60	4.79±0.96	1.68±1.24	1.53±0.30	1.05±0.28	1.44±0.55	2.97±0.86
F/Welch F	102.148	17.439	33.941	84.630	9.351	76.567
p	<0.001	<0.001	<0.001	<0.001	<0.001	<0.001
Gender						
Male	4.62±0.95	1.84±1.54	1.44±0.27	1.05±0.27	1.29±0.48	2.87±0.82
Female	4.49±0.91	1.54±1.09	1.50±0.28	0.95±0.27	1.42±0.49	2.69±0.79
t/t’	4.592	7.367	-7.339	11.873	-9.000	7.664
p	<0.001	<0.001	<0.001	<0.001	<0.001	<0.001
Occupation						
Agriculture/Forestry/Animal husbandry	4.71±0.91	1.69±1.32	1.53±0.29	1.03±0.27	1.45±0.61	2.91±0.81
Employees/Government officials	4.46±0.93	1.70±1.43	1.43±0.26	0.97±0.26	1.31±0.43	2.67±0.77
Others	4.50±0.93	1.62±1.20	1.47±0.27	0.98±0.28	1.36±0.46	2.74±0.81
F/Welch F	25.057	2.078	37.761	12.396	19.804	25.472
p	<0.001	0.125	<0.001	<0.001	<0.001	<0.001
Educational background						
Primary school or below	4.62±0.92	1.66±1.25	1.51±0.28	1.01±0.27	1.40±0.51	2.85±0.82
High school/Secondary vocational school	4.54±0.94	1.71±1.38	1.44±0.27	1.00±0.27	1.34±0.50	2.77±0.79
Junior college and above	4.23±0.84	1.49±1.20	1.40±0.27	0.86±0.26	1.35±0.38	2.40±0.71
F/Welch F	47.286	6.688	58.904	76.955	8.441	84.261
p	<0.001	0.001	<0.001	<0.001	<0.001	<0.001

Note: TC: Total cholesterol (mmol/L); TG: Triglyceride (mmol/L); HDL-C: High-density lipoprotein cholesterol (mmol/L); LDL-C: Low-density lipoprotein cholesterol (mmol/L); Apo A1 (g/L); Apo B(g/L).

### Prevalence of dyslipidemia

This study showed that the prevalence of HTC, HTG, low HDL-C and high LDL-C is 22.4%, 33.3%, 14.5%, 5.81%, respectively, and the overall prevalence of dyslipidemia in adults is 48.27% (Table 2).

**Table 2:** Prevalence of various types of dyslipidemia [Headcount (prevalence%)].

Item	Headcount	HTC	HTG	Low HDL-C	High LDL-C	Dyslipidemia
Prevalence		22.4	33.3	14.5	5.81	48.27
Area						
County seat	1817	423 (23.3)	630 (34.7)	223 (12.3)	97 (5.3)	890 (49)
Northern area (industrial)	1684	414 (24.6)	595 (35.3)	250 (14.8)	106 (6.3)	842 (50)
Southern area (rural)	1097	195 (17.8)	308 (28.1)	196 (17.9)	64 (5.8)	488 (44.5)
x^2^		18.895	18.134	17.403	1.463	8.681
p		<0.001	<0.001	<0.001	0.481	0.013
Age						
18~29	304	26 (8.6)	59 (19.4)	30 (9.9)	6 (2)	78 (25.7)
30~39	917	106 (11.6)	249 (27.2)	159 (17.3)	23 (2.5)	339 (37.0)
40~49	1287	254 (19.7)	435 (33.8)	210 (16.3)	72 (5.6)	615 (47.8)
50~59	1342	399 (29.7)	525 (39.1)	186 (13.9)	92 (7.2)	770 (57.4)
≥60	748	247 (33.0)	265 (35.4)	84 (11.2)	69 (9.2)	330 (55.9)
x^2^		190.559	64.108	21.475	47.445	171.207
p		<0.001	<0.001	<0.001	<0.001	<0.001
Gender						
Male	1840	464 (25.2)	707 (38.4)	391 (21.3)	140 (52.4)	1026 (55.8)
Female	2758	568 (20.6)	826 (29.9)	278 (10.1)	127 (47.6)	1194 (43.3)
x^2^		13.549	35.666	110.764	18.208	68.715
p		<0.001	<0.001	<0.001	<0.001	<0.001
Occupation						
Agriculture/Forestry/Animal husbandry	1055	310 (29.4)	352 (33.4)	151 (14.3)	81 (7.7)	565 (53.6)
Employees/Government officials	1189	224 (18.8)	404 (34.0)	206 (17.3)	48 (4.0)	556 (46.8)
Others	2354	498 (21.2)	777 (33.0)	312 (13.3)	138 (5.9)	1099 (46.7)
x^2^		40.311	0.335	10.595	13.574	15.245
p		<0.001	0.846	0.005	0.001	<0.001
Educational background						
Primary school or below	2328	566 (24.3)	780 (33.5)	313 (13.4)	161 (6.9)	1160 (49.8)
High school/Secondary vocational school	1695	395 (23.3)	593 (35)	290 (17.1)	92 (5.4)	859 (50.7)
Junior college and above	575	71 (12.3)	160 (27.8)	66 (11.5)	14 (2.4)	201 (35)
x^2^		39.061	9.959	15.579	17.633	47.016
p		<0.001	0.007	<0.001	<0.001	<0.001

Prevalence of dyslipidemia in different groups: (1) There is no difference for the prevalence of high LDL-C in different regions; other types of dyslipidemia are statistically significant. The prevalence of HTC and HTG in the county seat and northern rural area are significantly higher than that in the southern rural area; low HDL-C prevalence is different among regions, with the highest seen in the southern rural area. There are differences in the total prevalence of dyslipidemia, with the highest seen in the northern industrial area. (2) In the term of age, the difference of dyslipidemia is statistically significant. It was observed that the prevalence of HTC increases significantly with age in the range of 40–59 years old, but there is no difference between the groups aged under 39 and between the groups aged 50–59 and 60 above. Moreover, the prevalence of HTG and high LDL-C also increases with age, whereas the prevalence of low HDL-C peaks at 30–50 years old. The overall prevalence of dyslipidemia increases with age, but no longer after 60 years old. (3) In terms of gender, there are statistically significant differences between men and women, that is men have higher risks of dyslipidemia. (4) For the occupation, there is no difference in the prevalence of HTG among different groups, but the highest prevalence of low HDL-C was observed in Employees/Government official group, while the prevalence of high LDL-C and HTC as well as the overall prevalence of dyslipidemia are highest in Agriculture/Forestry/Animal husbandry group, and there is no difference in the other two occupations. (5) The differences of dyslipidemia among people with different educational backgrounds are statistically significant by the chi square test. Levels of HTC, HTG and the total prevalence of dyslipidemia are significantly higher for people with an education level below secondary vocational school. Low HDL-C level is mostly observed among people with a degree of high school or secondary vocational school or above (Table 2).

### Single-factor analysis for dyslipidemia

Factors including BMI, abdominal obesity, smoking or not, years of smoking, hypertension, blood glucose level, hyperuricemia, and cerebrovascular disease were analyzed for their effects on dyslipidemia in Shenmu City. As shown in Table 3, there is no statistically significant difference in the prevalence of dyslipidemia for factors like smoking amount, labor income and cardiovascular diseases, while the other influencing factors show significant effects on dyslipidemia prevalence. Firstly, the higher the BMI, the higher the incidence of dyslipidemia. Additionally, there is a statistically significant difference between non-smoking and smoking populations, regardless of whether one quits smoking or not now, suggesting that smoking is associated with dyslipidemia. Moreover, the duration of smoking history significantly influenced dyslipidemia, the longer the history of smoking, the higher the incidence of dyslipidemia. Comparing to the group smoking less than 10 years, the groups of 10–19 years, 20–29 years and >30 years displayed significantly higher prevalence. Then by analyzing relevance between blood glucose and dyslipidemia, it was revealed that abnormal glucose metabolism including prediabetes and diabetes displayed significantly higher incidence, while there is no significant difference between those with prediabetes and diabetes, investigating that abnormal glucose metabolism is associated with dyslipidemia. In addition, the prevalence of dyslipidemia is higher for those with abdominal obesity, hypertension, hyperuricemia, and cerebrovascular disease.

**Table 3:** Single-factor analysis results of dyslipidemia [Headcount (prevalence%)].

Influencing factor	Headcount	Dyslipidemia	x^2^	p
BMI			373.891	<0.001
<18.5	106	17 (16.0)		
18.5≤BMI<24	1805	602 (33.4)		
24≤BMI<28	1856	1039 (56.0)		
≥28	831	562 (67.6)		
Abdominal obesity	2215	1382 (62.4)	340.802	<0.001
Smoking or not			68.633	<0.001
Smoking at present	1353	757 (55.9)		
Smoking in the past	333	192 (57.7)		
Never smoke	2912	1271 (43.6)		
Smoking duration			16.194	0.001
Under 10 years	173	76 (43.9)		
10~19 years	322	190 (59.0)		
20~29 years	487	296 (60.8)		
More than 30 years	704	387 (55.0)		
Smoking amount (cigarettes/day)			3.395	0.183
<10	421	222 (52.7)		
10~19	428	240 (56.1)		
≥20	837	487 (58.2)		
Having labor income	2664	1292 (48.5)	0.119	0.730
Hypertension	1539	938 (60.9)	148.640	<0.001
Blood glucose			86.987	<0.001
Normal	4188	1932 (46.1)		
Prediabetes	128	89 (69.5)		
Diabetes	282	199 (70.6)		
Hyperuricemia	576	381 (66.1)	84.155	<0.001
Cardiovascular disease	240	130 (48)	3.512	0.061
Cerebrovascular disease	187	106 (56.4)	5.150	0.023

Hyperuricemia: Women, uric acid (UA)≥420 μmol/and men, UA≥360 μ mol/L; Cardiovascular disease: Participants have been diagnosed with myocardial infarction, angina pectoris, and coronary heart disease; Cerebrovascular disease: Participants have previously diagnosed with cerebral insufficiency, lacunar infarction, thrombosis, hemorrhage, and subarachnoid hemorrhage.

### Multivariate logistic regression analysis of factors influencing dyslipidemia

**Fig 2:**
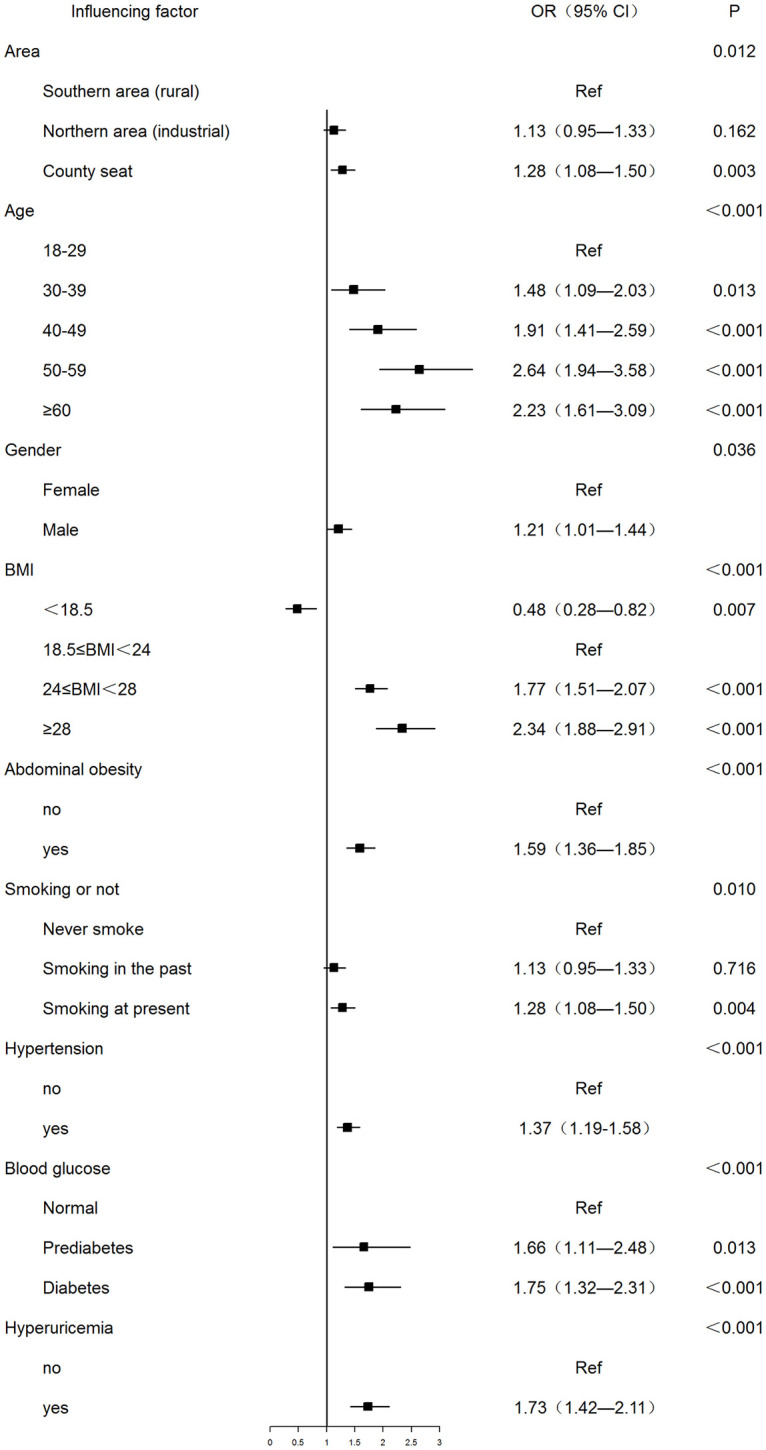
Multivariate logistic regression analysis of factors influencing dyslipidemia.

A total of 13 independent variables (confounding factors) in univariate analysis were included in the multivariate logistic regression model. There are 9 risk factors of dyslipidemia which had statistical significance-living in the county seat and northern industrial area, age increase, male, overweight or obesity, abdominal obesity, smoking, hypertension, abnormal glucose metabolism (pre-diabetes or diabetes), and hyperuricemia (Fig 2 and Supplemental information).

## Discussion

A survey in 2010 [13] on the blood lipid levels of Chinese adults showed that the average level of LDL-C was 3.93 mmol/L; for adults in western China, the TC average stood at 3.93 mmol/L, TG level at 1.35 mmol/L, HDL-C level at 1.09 mmol/L, and LDL-C at 2.17 mmol/L. According to the Report on Nutrition and Chronic Diseases of Chinese Residents in 2015 [14], the average TC level of Chinese adults was 4.5 mmol/L, TG level was 1.38 mmol/L, and HDL-C was 1.19 mmol/L. According to the Guidelines for Prevention and Treatment of Dyslipidemia in Chinese Adults in 2016 [3], the serum Apo A1 level was 1.2~1.6 g/L for normal Chinese population and remarkably positively correlated with HDL-C level. The Apo B of serum in normal population was 0.8~1.1 g/L, which mainly reflected LDL-C level, and correlated with LDL-C level.

Shenmu City is located in the northern part of Shaanxi Province, with an area of 7,635 km^2^. As the largest county-level city in Shaanxi Province, Shenmu has 14 towns, 6 sub-districts and 326 administrative villages under its jurisdiction, with a total population of 540,000. In 2019 it ranked 12^th^ in the list of China’s top 100 economically powerful counties [15]. However, this study revealed that the levels of TC, TG, and LDL-C of adults in Shenmu City are higher than the national averages and the levels in western China. The average level of HDL-C of Shenmu residents is lower than the national average, and the serum levels of Apo B and Apo A1 are in the normal range.

Distribution characteristics of blood lipid levels in this study indicated that, (1) the levels of TC and TG in the county seat and northern industrial area are higher than that in southern rural area, which might be a result of the differences in economic development between the north and the south of Shenmu City, considering that the county seat and the northern area are rich in coal and relatively well-off, while the southern area is scarce in resources and underdeveloped. The possible explanation might be higher income in the county seat and northern industrial area leads to more dining in restaurants and more consumption of the local delicacy-sheep entrails, which contain high cholesterol and fat contents. In comparison, residents of the agricultural area in the northern part mainly take self-produced vegetables and meat. Therefore, people in the county seat and northern industrial area have higher risk of dyslipidemia, and should examine blood lipid regularly for health. Besides, awareness of healthy lifestyle should be raised, and residents should be encouraged to consume more fresh vegetables and high-quality protein, and have a low-salt and low-fat diet [8,16]. (2) Higher levels of TC and LDL-C were observed among people working in Agriculture/Forestry/Animal husbandry. Most of those working in Agriculture/Forestry/Animal husbandry are directly engaged in coal-related industries, which bring them higher incomes. The labor force in southern rural area migrate to work in the energy industry in the north, which becomes their main source of income. It is evidenced by the higher levels of blood lipids among those in the occupation group of Agriculture/Forestry/Animal husbandry who do other jobs. This is the economic characteristics of Shenmu [17], which is of great value to guide the prevention and treatment of blood lipid disorders among people of different occupations in the region. (3) The levels of TC, TG, and LDL-C of men are generally higher than women, while HDL-C level is lower, which might be associated with the smoking habit, abdominal obesity, irrational diet and lack of exercise of men and consistent with previous findings [18–20]. (4) With increase of age, the levels of TC, TG and LDL-C increase and then decrease. The levels of TC and LDL-C peak in the age of 50 and TG level increases significantly after 40 years old. It is interesting to note that the levels of TC, TG and LDL-C no longer elevated significantly after 60 years old, which may be attributed to the use of medication, improvement in living standards, healthy eating habits, and more exercise. HDL-C level is lower at 30–49 years old, which indicated that age is an important factor to influence blood lipid levels, which is consistent with the findings of many studies from China and other countries [18,20]. Individualized prevention and treatment methods should be applied for patients with different age and blood lipid types. (5) The distribution of blood lipids is negatively correlated with the educational level: the higher the educational level, the higher the health awareness. Therefore, we should pay attention to health education among the people with lower educational levels, which account for 87.49% of the local population.

Results of a 2012 national survey [14] showed that the prevalence of HTC, HTG, low HDL-C among adults in China was 4.9%, 13.1% and 33.9%, respectively, and the overall prevalence of dyslipidemia among Chinese adults was 40.40%. According to the monitoring results of chronic diseases and their risk factors in China in 2013 [21], the prevalence of HTC, HTG, low HDL-C and high LDL-C was 5.1%, 14.3%, 23.6% and 6.1% respectively. In comparison with the findings of our study, the prevalence of HTC and HTG in Shenmu is notably higher than the average of China, and prevalence of low HDL-C and high LDL-C is relatively stable. In another providence (Liaoning Province) of China, Sun *et al* showed that 16.4%, 13.8%, 7.6% and 17.3% of local population had HTC, low HDL-C, high LDL-C and HTG concentrations, respectively and prevalence of lipid abnormality (including borderline dyslipidemia and dyslipidemia) was 47.8%, 13.8%, 25.7% and 30.7% for TC, HDL-C, LDL-C and TG [22]. Detailed analysis demonstrated that 36.9% of this population had at least one type of dyslipidemia and 64.4% had at least one type of abnormal lipid concentration [22]. In the US, the overall dyslipidemia prevalence (53%), prevalence of high LDL-C (27%) and low HDL-C (23%) of American [23] adults was lower than Shenmu adults, but the prevalence of HTG is higher than that the US (30%). Therefore, special attention should be given to prevention and control of HTC and HTG. In terms of distribution, the prevalence of HTC, HTG, low HDL-C and overall dyslipidemia in the county and northern industrial area is higher than that in southern rural area, with Agricultural/Forestry/Animal husbandry workers reporting the highest prevalence of HTC, high LDL-C, low HDL-C and overall dyslipidemia. These results indicate that dyslipidemia significantly increased with rapid economic growth, improved lifestyle changes and developed industry. The prevalence of dyslipidemia in men is higher than that for women. HTC prevalence peaks after the age of 50 and no longer significantly increases after 60. The prevalence of low HDL-C peaks between 30 to 50 years old. The prevalence of dyslipidemia is negatively correlated with educational level. These prevalence rates are consistent with the blood lipid levels. However, the prevalence of HTG continues to increase with age, even after 60 years old. Therefore, HTG level should be monitored frequently in the elderly population due to high risk of cardiovascular and cerebrovascular diseases.

The study found that the risk of dyslipidemia rises significantly with the increase of BMI. Overweight and obesity are important risk factors for dyslipidemia. Furthermore, we found that dyslipidemia is associated not only with overall fat, but also with increased abdominal fat, especially intra-abdominal fat. In our study, the risk of dyslipidemia for those with abdominal obesity is 1.60 times higher than normal people (95%CI: 1.37~1.87), which is consistent with previous reports [24,25]. At present, smoking is a definite independent risk factor for dyslipidemia. The time duration of smoking was always associated with the occurrence of dyslipidemia. It was found that the longer smoking history leads more severe the dyslipidemia. The severest dyslipidemia was observed in the population with a smoking history of more than 30 years old. Accumulating evidences [26] showed smoking can cause dyslipidemia and smoking increases the risk of metabolic syndrome [27]. Therefore, smoking should be stringently controlled to alleviate the increase of dyslipidemia. Hypertension, abnormal glucose metabolism, hyperuricemia and dyslipidemia often coexist, and affect each other. Disorder of blood glucose metabolism can lead to the imbalance of lipid anabolism and catabolism in the system, which further causes abnormal lipid metabolism. This is consistent with previous findings [28,29]. However, the limited sample size, inconsiderate contents of designed questionnaire, the potential impact of confounding factors (e.g. bad mood of participants, typo mistakes) and the introduction of biases might comprise the conclusion and need further validation.

This study is the first time to reveal the prevalence of dyslipidemia and associated risk factors among adult residents in Shenmu City, established the health database of adult population in this area, and offered a glimpse into the blood lipid status of northern Shaanxi area where coal mining is a pillar industry. This study provides scientific basis for study of the prevention and treatment of dyslipidemia in this area, and urge the local residents needs more urgent attention on dyslipidemia.

## Conclusion

In this study, it was illustrated that blood lipid levels and the prevalence of dyslipidemia are high among adults in Shenmu City, and vary by region, age, gender, occupation and educational level. Besides, living in county seat or northern industrial area, increasing age, male, overweight or obesity, abdominal obesity, smoking, hypertension, abnormal glucose metabolism (pre-diabetes or diabetes) and hyperuricemia are identified as risk factors of dyslipidemia. This result underlines an urgent need to develop routine screening and scientific intervention programs targeting HTC and HTG and control the risk factors such as obesity and hypertension through health awareness, healthy lifestyles and diet, which is beneficial to prevent cardiovascular and cerebrovascular diseases.

## Supporting information

S1 Checklist STROBE statement—Checklist of items that should be included in reports of observational studies. (DOCX)

S1 Table Multivariate logistic regression analysis of influencing factors of dyslipidemia. (DOCX)

S1 File (PDF)

S2 File (PDF)

S1 Data (RAR)
