# Supplementary material for: Prevalence of dyslipidemia and associated risk factors among adult residents of Shenmu City, China
Source: PLoS One. 2021 May 7;16(5):e0250573. doi: 10.1371/journal.pone.0250573 (PMC8104371; doi:10.1371/journal.pone.0250573)
Supplement: S1 Checklist — (DOCX) [file pone.0250573.s001.docx]

STROBE Statement—checklist of items that should be included in reports of observational studies

|  | Item No. | Recommendation | Page  No. | Relevant text from manuscript |
| --- | --- | --- | --- | --- |
| **Title and abstract** | 1 | (*a*) Indicate the study’s design with a commonly used term in the title or the abstract | 1 | a cross-sectional study |
|  |  | (*b*) Provide in the abstract an informative and balanced summary of what was done and what was found | 1 | Stratified multistage sampling;Questionnaire surveys and physical examinations were conducted, with SPSS26.0 used in statistical analysis.; |
| Introduction | | | |  |
| Background/rationale | 2 | Explain the scientific background and rationale for the investigation being reported | 1 | cardiovascular and cerebrovascular diseases are the principal reasons of death for Chinese people.preventing dyslipidemia is crucial to containing cardiovascular and cerebrovascular diseases. |
| Objectives | 3 | State specific objectives, including any prespecified hypotheses | 1 | provide scientific basis for the prevention and treatment of cardiovascular and cerebrovascular diseases. |
| Methods | | | |  |
| Study design | 4 | Present key elements of study design early in the paper | 1-2 | The research is based on a cross-sectional study of allergic diseases in the natural population of Shenmu City. |
| Setting | 5 | Describe the setting, locations, and relevant dates, including periods of recruitment, exposure, follow-up, and data collection | 1-2 | Shenmu City,Shenmu Hospital,from September 2019 to December 2019. |
| Participants | 6 | (*a*) *Cohort study*—Give the eligibility criteria, and the sources and methods of selection of participants. Describe methods of follow-up  *Case-control study*—Give the eligibility criteria, and the sources and methods of case ascertainment and control selection. Give the rationale for the choice of cases and controls  *Cross-sectional study*—Give the eligibility criteria, and the sources and methods of selection of participants | 2 | 4,706 people were surveyed, and the response rate was 94.1%. After excluding those without a laboratory blood lipid test result (n=108), 4,598 subjects were included in the analysis. |
|  |  | (*b*) *Cohort study*—For matched studies, give matching criteria and number of exposed and unexposed  *Case-control study*—For matched studies, give matching criteria and the number of controls per case |  |  |
| Variables | 7 | Clearly define all outcomes, exposures, predictors, potential confounders, and effect modifiers. Give diagnostic criteria, if applicable | 2 | Questionnaire survey,Physical examinations,Diagnostic criteria,Quality control. |
| Data sources/ measurement | 8* | For each variable of interest, give sources of data and details of methods of assessment (measurement). Describe comparability of assessment methods if there is more than one group | *2* | The research is based on allergic diseases in the natural population of Shenmu City.  The questionnaire was jointly designed by the Institute of Basic Medicine of the Chinese Academy of Medical Sciences.  Dyslipidemia according to the Guidelines for Prevention and Treatment of Dyslipidemia in Chinese Adults (2016 revised version);BMI;Abdominal obesity；Prediabetes |
| Bias | 9 | Describe any efforts to address potential sources of bias |  |  |
| Study size | 10 | Explain how the study size was arrived at | 2 | The sample of the study was estimated to be about 5,000 people |

Continued on next page

| Quantitative variables | 11 | Explain how quantitative variables were handled in the analyses. If applicable, describe which groupings were chosen and why |  |  |
| --- | --- | --- | --- | --- |
| Statistical methods | 12 | (*a*) Describe all statistical methods, including those used to control for confounding | 2-3 | Continuous variable blood lipid levels were described by mean and standard deviation, and the gender differences of blood lipid level were t-tested. The differences by region, age group, occupation and educational level were analyzed by variance analysis and compared in pairs. Counts of dyslipidemia were described by rate, chi-square test was used to compare differences between groups; and multivariate logistic regression was used to analyze the influencing factors of dyslipidemia. Tables were made using excel and R language. |
|  |  | (*b*) Describe any methods used to examine subgroups and interactions |  |  |
|  |  | (*c*) Explain how missing data were addressed |  |  |
|  |  | (*d*) *Cohort study*—If applicable, explain how loss to follow-up was addressed  *Case-control study*—If applicable, explain how matching of cases and controls was addressed  *Cross-sectional study*—If applicable, describe analytical methods taking account of sampling strategy |  |  |
|  |  | (*e*) Describe any sensitivity analyses |  |  |
| Results | | | | |
| Participants | 13* | (a) Report numbers of individuals at each stage of study—eg numbers potentially eligible, examined for eligibility, confirmed eligible, included in the study, completing follow-up, and analysed | 3 | Among the 4,598 respondents, 1,817 live in the county seat, 1,097 in the southern rural area, and 1,684 in northern industrial area. |
|  |  | (b) Give reasons for non-participation at each stage |  |  |
|  |  | (c) Consider use of a flow diagram |  |  |
| Descriptive data | 14* | (a) Give characteristics of study participants (eg demographic, clinical, social) and information on exposures and potential confounders | 3 | The average age of the respondents is 47.43±11.31 years, 99.87% of the total. 4,258 respondents were born in Shenmu City, making up 92.60% of the total. 4,567 people now live in Shenmu City, accounting for 99.33% of the total. The occupations of the respondents were categorized as Agriculture/Forestry/Animal husbandry workers, Employees/Government officials, and Others (self-employed, unemployed, students, etc.). Education background: primary school or below (2,328 people), secondary school or secondary vocational school (1,695 people), junior college or above (575). Marital status: 4,292 are married, accounting for 93.34%. |
|  |  | (b) Indicate number of participants with missing data for each variable of interest |  |  |
|  |  | (c) *Cohort study*—Summarise follow-up time (eg, average and total amount) |  |  |
| Outcome data | 15* | *Cohort study*—Report numbers of outcome events or summary measures over time |  |  |
|  |  | *Case-control study—*Report numbers in each exposure category, or summary measures of exposure |  |  |
|  |  | *Cross-sectional study—*Report numbers of outcome events or summary measures | *3-8* | Average value of blood lipids ;Prevalence of dyslipidemia; Single-factor analysis for dyslipidemia;Multivariate logistic regression analysis of influencing factors of dyslipidemia |
| Main results | 16 | (*a*) Give unadjusted estimates and, if applicable, confounder-adjusted estimates and their precision (eg, 95% confidence interval). Make clear which confounders were adjusted for and why they were included |  |  |
|  |  | (*b*) Report category boundaries when continuous variables were categorized |  |  |
|  |  | (*c*) If relevant, consider translating estimates of relative risk into absolute risk for a meaningful time period |  |  |

Continued on next page

| Other analyses | 17 | Report other analyses done—eg analyses of subgroups and interactions, and sensitivity analyses |  |  |
| --- | --- | --- | --- | --- |
| Discussion | | | | |
| Key results | 18 | Summarise key results with reference to study objectives | 8-10 | This study showed that the levels of TC, TG, and LDL-C of adults in Shenmu City are higher than the national averages and higher than levels in western China. The average level of HDL-C of Shenmu residents is lower than the national average, and the serum Apo B, Apo A1 levels are in the normal range.  Distribution characteristics of blood lipid levels in this study showed that, (1) the levels of TC and TG in the county seat and northern industrial area are higher than that in southern rural area. |
| Limitations | 19 | Discuss limitations of the study, taking into account sources of potential bias or imprecision. Discuss both direction and magnitude of any potential bias | 8-10 | As shown in the manuscript |
| Interpretation | 20 | Give a cautious overall interpretation of results considering objectives, limitations, multiplicity of analyses, results from similar studies, and other relevant evidence | 8-10 | As shown in the manuscript |
| Generalisability | 21 | Discuss the generalisability (external validity) of the study results | 8-10 | This study for the first time revealed the blood lipid levels, distribution characteristics and influencing factors for adults in Shenmu City, established the health big data of natural population in this area, and offered a glimpse into the blood lipid status of northern Shaanxi area where coal mining is a pillar industry. It provides scientific basis for study of the prevention and treatment of dyslipidemia in this area. |
| Other information | |  | | |
| Funding | 22 | Give the source of funding and the role of the funders for the present study and, if applicable, for the original study on which the present article is based |  | Shenmu Municipal Government Scientific Research Project (2019) No.5 |

*Give information separately for cases and controls in case-control studies and, if applicable, for exposed and unexposed groups in cohort and cross-sectional studies.

**Note:** An Explanation and Elaboration article discusses each checklist item and gives methodological background and published examples of transparent reporting. The STROBE checklist is best used in conjunction with this article (freely available on the Web sites of PLoS Medicine at http://www.plosmedicine.org/, Annals of Internal Medicine at http://www.annals.org/, and Epidemiology at http://www.epidem.com/). Information on the STROBE Initiative is available at www.strobe-statement.org.
