# Supplementary material for: Prevalence of dyslipidemia and associated risk factors among adult residents of Shenmu City, China
Source: PLoS One. 2021 May 7;16(5):e0250573. doi: 10.1371/journal.pone.0250573 (PMC8104371; doi:10.1371/journal.pone.0250573)
Supplement: S1 Table — (DOCX) [file pone.0250573.s002.docx]

Multivariate logistic Regression Analysis of Influencing Factors of Dyslipidemia

| Influencing factor | β | Wald x^2^ | OR（95%CI） | p |
| --- | --- | --- | --- | --- |
| Area |  |  |  |  |
| Southern area (rural) |  |  | 1.00 |  |
| Northern area (industrial) | 0.119 | 1.957 | 1.13（0.95—1.33） | 0.162 |
| County seat | 0.244 | 8.548 | 1.28（1.08—1.50） | 0.003 |
| Age |  |  |  |  |
| 18～29 |  |  | 1.00 |  |
| 30～39 | 0.394 | 6.128 | 1.48（1.09—2.03） | 0.013 |
| 40～49 | 0.646 | 17.360 | 1.91（1.41—2.59） | ＜0.001 |
| 50～59 | 0.969 | 38.640 | 2.64（1.94—3.58） | ＜0.001 |
| ≥60 | 0.801 | 23.223 | 2.23（1.61—3.09） | ＜0.001 |
| Gender |  |  |  |  |
| Female |  |  | 1.00 |  |
| Male | 0.190 | 4.399 | 1.21（1.01—1.44） | 0.036 |
| BMI |  |  |  |  |
| ＜18.5 | -0.744 | 7.198 | 0.48（0.28—0.82） | 0.007 |
| 18.5≤BMI＜24 |  |  | 1.00 |  |
| 24≤BMI＜28 | 0.569 | 50.780 | 1.77（1.51—2.07） | ＜0.001 |
| ≥28 | 0.849 | 57.570 | 2.34（1.88—2.91） | ＜0.001 |
| Abdominal obesity |  |  |  |  |
| no |  |  | 1.00 |  |
| yes | 0.461 | 33.791 | 1.59（1.36—1.85） | ＜0.001 |
| Smoking or not |  |  |  |  |
| Never smoke |  |  | 1.00 |  |
| Smoking in the past | 0.052 | 0.132 | 1.13（0.95—1.33） | 0.716 |
| Smoking at present | 0.276 | 8.281 | 1.28（1.08—1.50） | 0.004 |
| Hypertension |  |  |  |  |
| no |  |  | 1.00 |  |
| yes | 0.318 | 19.324 | 1.37（1.19-1.58） | ＜0.001 |
| Blood glucose |  |  |  |  |
| Normal |  |  | 1.00 |  |
| Prediabetes | 0.506 | 6.119 | 1.66（1.11—2.48） | 0.013 |
| Diabetes | 0.557 | 15.256 | 1.75（1.32—2.31） | ＜0.001 |
| Hyperuricemia |  |  |  |  |
| no |  |  | 1.00 |  |
| yes | 0.547 | 29.008 | 1.73（1.42—2.11） | ＜0.001 |
