# Supplementary material for: Prevalence of dyslipidemia and associated risk factors among adult residents of Shenmu City, China
Source: PLoS One. 2021 May 7;16(5):e0250573. doi: 10.1371/journal.pone.0250573 (PMC8104371; doi:10.1371/journal.pone.0250573)
Supplement: S1 File — (PDF) [file pone.0250573.s003.pdf]

编号: ____________ 日期: ____________ 受访者姓名:___________ 调研员姓名: _____________

神木市成人血脂分布特征及影响因素分析调查表

医院名称: __ __ __ 医院所在地: __ __ __ 病历号: __ __
受访者编号(ID): __ __ __ __ __ 调查日期: _ _年_ _月_ _日

A.一般情况

01.受访者姓名 ________________ 02.性别: 男=1 女=2

03.年龄__ __ 岁 04.出生日期 __ __ 年__ __ 月__ __ 日

05.您的户口是否在此地 是=1 否=2 06.出生地 __ __ __省__ __ __(市) __ __ __ (县)

07.本地居住年限__ __ 年

08.民族 汉=1 其他=2

09.职业(离退休前) 国家机关/党群组织/企业/事业单位负责人＝1 专业技术人员＝2 办事人员和有关人员＝3
商业/服务业人员＝4 农/林/牧/渔/水利业生产人员＝5 生产/运输设备操作人员及有关人员＝6
军人＝7 其他＝8

10 a.教育水平 硕士研究生毕业及以上＝1 大学本科/专科毕业＝2 普通高级中学/中等职业毕业＝3
初级中学毕业＝4 小学毕业＝5 其他＝6

b.在学校受教育年数: __ __ 年

11.婚姻状况 未婚=1 已婚=2 分居=3 离婚=4 丧偶=5

12.经济收入 a.家庭人口 __ __人
b.劳动收入 有＝1 无＝0 退休工资 有＝1 无＝0 子女赡养 有＝1 无＝0 其他
有＝1 无＝0
c.您现在是如何生活的: 独居=1, 与配偶居住=2, 与子女和配偶居住=3, 与子女居住=4,
与其他人同住=5

13. 您是否吸烟?

a. 您是否吸烟?
现在吸烟
曾经吸烟
从不吸烟

b. 您开始吸烟的年龄: __ __ 岁
若已戒烟,戒烟年龄: __ __ 岁

c. 您目前或在戒烟前通常吸哪类烟,具体数量是多少?(如习惯混吸,最多可同时选择三项主要的回答)

机制卷烟=1	支/天=1
手卷烟、旱烟=2	两/月=2
烟斗、水烟=3	两/月=3
雪茄 =4	支/天 =4
其他 =5	

14.家庭地址 __________ 市, __________ 区(县), ___________ 街(乡), ___________ 居委会(村)
__________ 门牌号, 邮编 _________ 电话号码(家) ________ (办公室) ________

15.工作单位和地址____; 邮编 __________

16.您过去或目前是否有下列疾病?

访员:假如受访者回答是,则继续问: "看过病吗,是否有病例纪录?"
有,且有病历记录=1 有,但无病历记录=2 无=0

编号: ____________ 日期: ____________ 受访者姓名:___________ 调研员姓名: _____________

a 肺气肿或慢性支气管炎,哮喘
b 心律不齐
c 心肌梗塞,心绞痛
d 其它类型心脏病 (指明_______________)
e 高血压
f 肝硬化
g 肾脏疾病 (指明_______________)
h 糖尿病
i 甲状腺功能低减
j 甲状腺功能亢进
k 恶性肿瘤 (指明_______________)
l 脑外伤
m 脑血管病(腔梗,血栓,出血,蛛网膜下出血)
z 其它疾病

n 脑供血不足
o 运动神经元病
p 共济失调
q 癫痫
r 高脂血症
s 酒依赖或中毒
t 精神疾病 (指明_______________)
u 白内障
v 耳聋(需大声说话或用助听器)
w 前列腺肥大或小便不畅
x 骨关节畸形(指,趾,脊柱,其他___)
y 骨折*(股骨,上肢,其他 _________)
* 是指发生在50岁以后的骨折

体格检查 体检时间: 来源:

包括:
身高、体重、血压(收缩压/舒张压 mmHg)、腰围、体重指数(BWI)
血常规
尿常规
肝功能
糖脂
心电图
胸片

访员:姓名: ______________ 访员的性别: ①男; ②女 工作单位:________________
职业身份: ①大学生 ②护士 ③其他 ______ 签名: _________

复查人:姓名: ______________ 工作单位:_____________________ ____年___ 月 ___日
职业身份: ①大学生 ②护士 ③其他 ______ 签名: _________

最后复核人:姓名: ______________ 工作单位:_____________________ 签名: _________
