# Supplementary material for: Prevalence of dyslipidemia and associated risk factors among adult residents of Shenmu City, China
Source: PLoS One. 2021 May 7;16(5):e0250573. doi: 10.1371/journal.pone.0250573 (PMC8104371; doi:10.1371/journal.pone.0250573)
Supplement: S2 File — (PDF) [file pone.0250573.s004.pdf]

Number:_______ Date:_______ Name of interviewee:______ Name of researcher:_______

Questionnaire on distribution characteristics and influencing factors of adult blood lipid in Shenmu city

Hospital name:_ _ Hospital Location:_ _ Medical Record Number:_ _
Interviewee Number (ID):_ _ Investigation Date:_ _

A.General situation

01.Name of interviewee _ _ 02.Gender: male =1 female =2

03.Age _ _ years old 04.Date of birth _ _year_ _month_ _day

05.Is your account here Yes=1 No =2 06.Place of birth _ _ province_ _ (city) _ _ (county)

07.Local residence time _ _ years

08.Ethnic Han =1 others =2

09.Occupation (before retirement) Head of state organs/party-mass organizations/enterprises/institutions = 1
Professional and technical personnel = 2 Clerks and relevant personnel = 3
Commercial/service personnel = 4 Agricultural/forestry/animal husbandry/fishery/water conservancy production personnel = 5
Production/transportation equipment operators and related personnel = 6
Soldiers= 7 Others = 8

10.a.Education level Master's degree or above = 1 Undergraduate/junior college graduation = 2
Ordinary high school/secondary vocational graduation = 3
Junior high school graduation = 4 Primary school graduation = 5 Others = 6

b.Years of schooling:_ _ years

11.marital status unmarried =1 married =2 separated =3 divorced =4 widowed =5

12.Economic income a.the family population is _ _people
b.Labor income Yes =1 None = 0 Retirement salary Yes = 1 None = 0 Children support Yes = 1 None = 0 Others Yes = 1 None = 0
c.How do you live now: living alone =1, living with your spouse =2, living with children and spouses =3, living with children =4, Living with others =5

13.Do you smoke?

a.Do you smoke?
Smoking now
Used to smoke
Never smoke

b.The age at which you started smoking:_ _ years old
If you have quit smoking,the age of quitting smoking is _ _ years old

c.What kind of cigarettes do you usually smoke at present or before you quit smoking, and what is the specific quantity? (If you are used to mixed smoking, you can choose up to three main answers at the same time)
Machine cigarette =1 cigarette/day =1
Hand cigarettes, dry cigarettes =2 two/month =2
Pipe, hookah =3 two/month =3
Cigars =4 cigarettes/day =4
Other =5

14.The home address is _ _ City, _ _ district (county), _ _ street (township), _ _ neighborhood Committee (village) _ _ house number, zip code _ _ telephone number (home) _ _ (office) _ _

Number:_______ Date:_______ Name of interviewee:______ Name of researcher:_______

15.Work unit and address _ _; zip code _ _

16.Do you have the following diseases in the past or at present?

Interviewer: If the interviewee answers yes, continue to ask: "Have you ever seen a doctor? Is there a case record?"

Yes,with medical records =1 yes,but no medical records =2 no =0

a emphysema or chronic bronchitis, asthma
b arrhythmia
c myocardial infarction, angina pectoris
d other types of heart disease (specify _ _)
e hypertension
f cirrhosis
g kidney disease (specify _ _)
h diabetes
i hypothyroidism
j hyperthyroidism
k malignant tumor (specify _ _)
l traumatic brain injury
n cerebral blood supply insufficiency
o motor neuron disease
p ataxia
q epilepsy
r hyperlipidemia
s alcohol dependence or poisoning
t mental disease (specify _ _)
u cataract
v deafness (need to speak loudly or use hearing AIDS)
w prostatic hypertrophy or dysuria
x bone and joint deformity (finger,toe,spine,other _ _)
y fracture * (femur,upper limb,other _ _)
* refers to fractures occurring after the age of 50
m cerebrovascular disease (lacunar infarction, thrombosis, hemorrhage, subarachnoid hemorrhage)
z other diseases

Physical examination time: Source:
Include:
Height, weight, blood pressure (systolic blood pressure/diastolic blood pressure mmHg), waist circumference, body mass index (BWI)
routine blood test
Urine routine
liver function
glycolipid
electrocardiogram
chest radiograph

Interviewer: Name: _ _ Interviewer's gender: ① Male; ② Female work unit: _ _
Professional identity: ① College student ② Nurse ③ Other _ _ Signature: _ _

Reviewed by: Name: _ _ Work unit: _ _ _ _year_ _month_ _day
Professional identity: ① College student ② Nurse ③ Other _ _ Signature: _ _

Final reviewer: name: _ _ work unit: _ _
